# NEED FOR SUPPORT FOR PERSONS WITH DISABILITIES IN ELDER ABUSE

**DOI:** 10.1093/geroni/igac059.1360

**Published:** 2022-12-20

**Authors:** Hitoshi Suda, Tadashi Wada, Kana Satoh

**Affiliations:** SEITOKU University, Matsudo city, Chiba, Japan; Irahara Primary Care Hospital, Matsudo City, Chiba, Japan; Teikyo University of Science, Adachi, Tokyo, Japan

## Abstract

Among the elder abuse cases reported to Matsudo City, Chiba Prefecture (population 487,091 as of April 1, 2017) from fiscal year (FY) 2017 to FY 2019. Of the 525 reported cases, 497 for which records could be judged to be accurate were included in the study. There were 119 cases (23.9%) in which the abusers were presumed to have a disability. Out of these 119 cases, the abusers of 86 (72.3%) cases were not involved with health and welfare policies for people with disabilities or such involvement had been suspended. If the abuser had received support in the field of health and welfare for the disabled or as a person in need at an early stage, the elder abuse might not have occurred. Support for people with disabilities and support for persons in financial need are required before they become abusers.

